# Association between IL28B Polymorphisms and Outcomes of Hepatitis B Virus Infection: A meta-analysis

**DOI:** 10.1186/s12881-020-01026-w

**Published:** 2020-05-01

**Authors:** Jingyu Zhao, Xinyue Zhang, Liwei Fang, Hong Pan, Jun Shi

**Affiliations:** 1 State Key Laboratory of Experimental Hematology, National Clinical Research Center for Blood Diseases, Institute of Hematology & Blood Diseases Hospital, Chinese Academy of Medical Sciences & Peking Union Medical College, Regenerative Medicine Clinic, Tianjin, 300020 China; 2 Tianjin Medical University Cancer Institute and Hospital, National Clinical Research Center for Cancer, Key Laboratory of Cancer Prevention and Therapy, Tianjin, Tianjin’s Clinical Research Center for Cancer, Tianjin, 300060 China

**Keywords:** Interleukin 28B polymorphisms, Persistent HBV infection, Hepatitis B virus-related hepatocellular carcinoma, Meta-analysis

## Abstract

**Background:**

Interleukin (IL) *28B* polymorphisms encoding pro-inflammatory and anti-inflammatory cytokines trigger diverse clinical outcome of hepatitis virus infection. However, there is controversy concerning the association of *IL28B* polymorphisms with the outcome of hepatitis B virus (HBV) infection, with several studies obtaining inconsistent results. We performed a meta-analysis to evaluate the role of 3 single nucleotide polymorphisms (SNPs) rs12979860, rs12980275 and rs8099917 in the progression of HBV infection, overall and by ethnicity.

**Methods:**

Searched PubMed, Embase and Wiley Online Library electronic databases using ‘interleukin 28B’, ‘IL 28B’, ‘IL 28B polymorphism’, ‘hepatitis B virus’, ‘HBV’, and performed meta- analysis for rs12979860, rs12980275 and rs8099917 in Asian and Caucasian populations under the dominant recessive and allele model.

**Results:**

Eighteen studies were found in total and used for this meta-analysis, including 5587 cases and 4295 controls. The *IL28B* polymorphism rs12979860 had no association with HBV persistence (CC vs CT + TT: OR = 0.86, 95% CI = 0.76–1.00; TT vs CT + CC: OR = 1.14, 95% CI = 0.76–1.70; T vs C: OR = 1.03, 95% CI = 0.94–1.13). Similarly, neither rs12980275 nor rs8099917 had associations with HBV persistence (rs12980275 in AA vs AG + AA: OR = 1.15, 95% CI = 0.96–1.38; rs8099917 in TT vs GT + GG: OR = 1.15, 95% CI = 0.96–1.39). There was also no significant association of *IL28B* polymorphisms with persistent HBV infection in Asians or Chinese. There was no evidence of an association of rs12979860 with the HBV-related hepatocellular carcinoma susceptibility (T vs C: OR = 1.53, 95% CI = 0.96–2.43).

**Conclusion:**

*IL28B* polymorphisms had no association with the outcome of HBV infection overall, nor in the Asians and the Chinese. These 3 SNPs might not be relevant to the development of HBV infection.

## Background

Hepatitis B virus (HBV) is a serious public health issue which contributes to the global burden of disease. According to the World Health Organization, HBV claimed 887,000 lives in 2015 and resulted in 257 million chronic carriers in 2017 [1]. Moreover, approximately 2.7 million chronic carriers are also infected with human immunodeficiency virus (HIV) because HBV and HIV can both be transmitted by unprotected sex and injection drug use [2], living conditions that preclude access to running water [3], as well as from mother to child during pregnancy and birth [4]. Approximately 2 million disability-adjusted life-years (DALYs) are attributed to HBV infection [5]. However, the global burden of chronic HBV is unequally distributed by region. Incidence is high in Africa and Asia, and the endemic high prevalence of HBV and hepatitis C virus (HCV) infection is attributed to persistent infection [6]. HBV infection contributes a greater burden of disease in many developing countries. A safe and effective vaccine has been available since the 1980s, however, vaccination strategies differ from country to country due to cost [7]. It is still a great challenge for developing countries to prevent HBV infection.

HBV is a contagious liver disease that can be acute or chronic. Some people who get HBV recover completely from the acute illness, while others may develop a lifelong infection, which increases the risk of liver cirrhosis, liver failure and hepatitis hepatocellular carcinoma (HHC). HCC is the third leading cause of cancer death globally [8], and 78% of HCC is attributed to HBV or HCV [5]. The natural history of HBV infection varies from spontaneous recovery post-infection, to chronic asymptomatic carrier, to decompensated cirrhosis and liver cancer [9]. However, the underlying biological mechanisms that induce the different outcomes of HBV infection remain to be discovered, and hopefully exploited as a target of intervention or incorporated into a risk prediction tool.

Cytokines and regulatory molecules made a major contribution to the immune-pathogenesis of HBV infection. The cytokine Interferon-λ3 (*IFN*-λ3) coded by interleukin (*IL*) *28B* polymorphisms has an anti-viral effect and could impede the HBV replication in hepatocyte cell lines. In recent years, several genome-wide association studies (GWAS) indicate the 3 single-nucleotide polymorphisms (SNPs) rs12979860 C/T, rs12980275 A/G and rs8099917 T/G, located on *IL28B* are associated with liver diseases [10, 11]. Furthermore, *IL28B* polymorphisms predict the serological response to Pegylated interferon-α (*PEG-IFN*-α) in terms of HBeAg sero-conversion that relate to HBV infection [12]. The association of genotypic variations in *IL28B* with HBV infection suggests a potential therapeutic target.

Currently, associations of *IL28B* with HBV infection are not completely consistent. For instance, the SNP rs12979860 was reported to be strongly related to HBV persistence under the allelic and dominant models [13]. Conversely, Song found there was no association of rs12979860 with the outcome of HBV infection [14]. In addition, several studies suggest a strong association of the *IL28B* gene with the HBV/HCV-induced HCC [15–17]. Nevertheless, few studies have specifically explored the relationship of the *IL28B* gene with HBV-related HCC.

## Methods

### Search strategy

We followed the PRISMA guidelines to perform this systematic review and meta-analysis. A systematic research of PubMed, Embase, Wiley Online Library databases was made with restriction to the English language from January 1, 2010 to June 1, 2018. The search terms included ‘interleukin 28B’, ‘IL 28B’, ‘IL 28B polymorphism’, and these terms in combination with ‘hepatitis B virus’ or ‘HBV’. Reference lists of the identified studies were also searched manually for additional eligible studies.

### Selection criteria

The inclusion criteria were as follows: (i) studies of persistent HBV infection patients, i.e. chronic carriers with chronic hepatitis or liver cirrhosis or hepatocellular carcinoma as cases, and healthy participants without HBV infection or HBV recovered patients as controls; (ii) studies with precise *IL28B* genotypes in case and controls; (iii) studies providing odds ratios (OR) and 95% confidence intervals (CI) for the dominant model (CC vs CT + TT for rs12979860; AA vs AG + GG for rs12980275; TT vs GT + GG for rs8099917), recessive model (TT vs CT + CC for rs12979860; GG vs AG + AA for rs12980275; GG vs GT + TT for rs8099917), and allelic model (T vs C for rs12979860; G vs A for rs12980275; G vs T for rs8099917); (iv) case-control study design; (v) diagnosis of chronic HBV carriers based on seropositive results for hepatitis B surface antigen (HBsAg) for more than 6 months; diagnosis of HBV recovery based on seropositive results for hepatitis B core antibody (anti-HBc) and hepatitis B surface antibody (anti-HBs) without HBsAg for at least 6 months. The exclusion criteria were: (i) studies lacking healthy controls or HBV recovered controls; (ii) studies with inaccurate or insufficient information on *IL28B* genotypes and the genetic models of interest; (iii) studies not designed as a case-control study; (iv) studies including participants testing positive for antibodies against HCV and HIV.

### Data extraction and quality assessment

We reviewed and identified all relevant publications according to the selection criteria, and extracted all available data for the eligible studies. The extracted information for each study included: name of the first author, year of publications, country of origin, ethnicity, SNP, number of genotyped cases and controls, and *P*-value derived from Hardy-Weinberg equilibrium (HWE) in controls.

The quality of each study was evaluated based on the Newcastle-Ottawa Scale (NOS), and 7–9 stars was perceived as high quality [18]. The appraisal items included factors, such as the comparability and representativeness of participants; ascertainment method of exposure; ascertainment method of cases and controls.

### Statistical analysis

We assessed the association of *IL28* polymorphisms rs12979860, rs12980275, rs8099917 with persistent HBV infection from which we reported ORs and 95% CIs from 3 genetic models. We also compared HCC patients as cases with healthy controls or recovery controls, used ORs and 95% CIs derived from the allelic model to examine the association of rs12979860 with the occurrence of HBV-related HCC. Stratified analysis was performed by ethnicity. Heterogeneity between studies was assessed by the chi-square-based Cochran’s Q-test and Higgins (*I*^*2*^) statistics, and the significance level was set at *P* = 0.1. Sequential omission of individual studies was conducted as a sensitivity analysis to test the heterogeneity. The impact of the 3 SNPs throughout meta-analysis was combined using a fixed-effects model (*P* ≥ 0.1 and *I*^*2*^ ≤ 0.25) and a random-effects model (*P* < 0.1 or *I*^*2*^ > 0.25). Visual inspection of asymmetry in the funnel plot was used to assess publication bias. Furthermore, Begg’s test and Egger’s test were also performed as a sensitivity analysis to examine publication bias. A *P*-value < 0.05 indicated publication bias was significant across studies. Trim and fill analysis was also conducted as a sensitivity analysis to diagnose publication bias in this meta-analysis. The statistical analysis was conducted by using R software, Version 3.3.3.

## Results

### Study characteristics

**Fig. 1:**
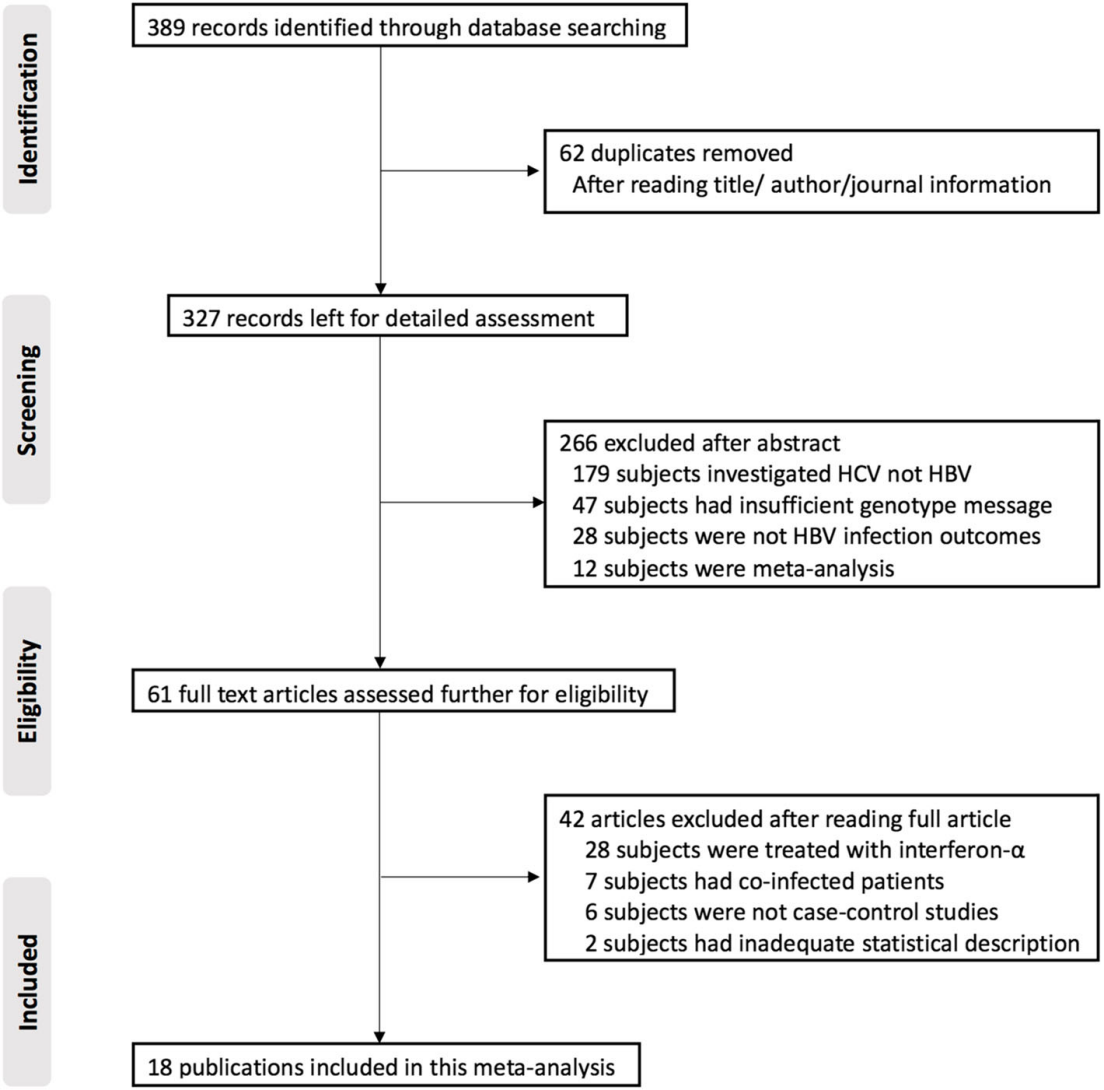
Flow-chart of literature search. HBV: hepatitis B virus; IL28B: interleukin 28B

The study selection and inclusion process are illustrated in Fig. 1. A total of 389 publications were identified by the search, and 62 duplicates were removed based on title, author and journal information. This gave 327 papers for detailed assessment from which 266 publications were excluded. Among those excluded articles, 179 publications focused on HCV infection rather than HBV; 47 publications lacked precise information on genotype; 28 publications lacked relevant HBV infection outcome and 12 publications were meta-analysis. The remaining 61 publications were assessed based on the above selection criteria, and 42 were excluded. Among them, 28 publications concentrated on interferon-α treatment, 8 publications recruited co-infected participants, and 6 publications were cohort studies. Finally, 18 full published studies were included in this systematic review and meta-analysis after this screening.

These 18 publications (Table 1) were published from 2010 to 2017, with the language restricted to English. There were 11 studies in Asia populations including Chinese Han, Korean and Thais, and the other 8 studies focused on other groups including American, Iranian, Italian, Spanish, Saudi Arabian and Turkish. All studies used persistent HBV infection patients as cases, and used healthy participants or patients recovered from prior HBV infection as controls. The 3 SNPs were genotyped using PCR, PCR-RELP or TaqMan assays. Minor allele frequencies of these 3 SNPs in Asians were significantly lower than those of the Caucasians (Additional file 1). All studies were hospital-based or population-based case-control studies. In total the 18 studies had 5587 cases and 4295 controls. There were 15 studies comprising 4913 cases and 3865 controls that considered rs12979860 C/T; 8 studies comprising 3765 cases and 2506 controls that considered rs12980275 A/G; and 11 studies comprising 3912 cases and 2900 controls that considered rs8099917 T/G. The distribution of genotypes in the controls of all studies was in agreement with HWE except for the study of Ren S [19]. The median NOS score of these studies was 7, and all studies had a high methodological quality according to NOS score. As shown in Table 1, 12 studies obtain 7 stars and 6 studies obtain 8 stars. Majority of the 8-star publications had well-matched cases and controls for gender, HBV copies and other relative factors. The common of most 7-star publications was that their data sources were mainly from the hospital, which might also lead to selection bias. In addition, some of them failed to fulfill the standard of comparability, i.e. study controls lack of description on matching for an additional factor.

**Table 1:** Characteristics of included studies in the meta-analysis

Author	Years/countries	Ethnicity	Sources	SNPs	Genotypes	Sample size			HWE in control	NOS score
						Healthy control	HBV recovery	HBV persistence		
Martin MP [20]	2010/United States	Caucasian	Hospital-based	rs12979860	CC/CT/TT T vs C	–	157/175/52 279 vs 489	99/94/33 160 vs 292	0.77	7
L.J.Peng [21]	2011/China	Asian	Population-based	rs12979860	CC/CT + TT	–	206/22	574/77	> 0.05	8
Li WY [22]	2011/China	Asian	Hospital-based	rs12979860	CC/CT + TT	179/24	180/23	178/25	> 0.05	8
				rs12980275	AA/AG + GG	178 vs 25	181/22	179/24	> 0.05	
				rs8099917	TT/GT + GG	182/21	182/21	180/23	> 0.05	
Fabris C [23]	2011/Italy	Caucasian	Population-based	rs12979860	CC/CT/TT T vs C	164/145/35 215 vs 473	–	36/35/4 43 vs 107	0.386	8
Chen J [24]	2012/China	Asian	Population-based	rs12979860	CC/CT/TT T vs C	213/29/2 33 vs 455	–	1043/152/10 172 vs 2238	0.37	8
				rs12980275	AA/AG/GG G vs A	210/33/1 35 vs 453	–	1036/162/8 178 vs 2234	> 0.05	
				rs8099917	GG/GT/TT G vs T	218/25/1 27 vs 461	–	1075/126/4 130 vs 2276	> 0.05	
Ren S [19]	2012/China	Asian	Hospital-based	rs12979860	CC/CT/TT T vs C	43/4/0 4 vs 90	33/7/3 13 vs 73	177/46/16 78 vs 400	0.01	8
				rs12980275	AA/AG/GG G vs A	43/3/1 5 vs 89	37/5/1 7 vs 79	205/30/4 38 vs 440	0.01	
				rs8099917	GG/GT/TT G vs T	44/2/1 4 vs 90	33/6/4 14 vs 72	197/31/11 53 vs 425	0.07	
Luz MC [25]	2012/Spain	Caucasian	Hospital-based	rs12979860	CC/CT/TT T vs C	–	22/21/6 33 vs 65	29/17/3 23 vs 75	0.78	7
Lee DH [26]	2013/South Korea	Asian	Hospital-based	rs12979860	T vs C	–	24 vs 380	62 vs 973	> 0.05	7
				rs12980275	G vs A	–	27 vs 377	70 vs 965	> 0.05	
				rs8099917	G vs T	–	22 vs 382	56 vs 979	> 0.05	
Al-Qahtani [27]	2013/Saudi Arabia	Caucasian	Hospital-based	rs12979860	CC/CT/TT T vs C	–	174/91/20 131 vs 439	450/274/92 458 vs 1174	> 0.05	7
				rs12980275	AA/AG/GG G vs A	–	175/93/36 165 vs 443	548/229/44 317 vs 1325	> 0.05	
				rs8099917	GG/GT/TT G vs T	–	15/73/216 103 vs 505	31/187/606 249 vs 1399	> 0.05	
Kim SU [13]	2013/South Korea	Asian	Hospital-based	rs12979860	CC/CT/TT T vs C	85/15/0 15 vs 185	189/31/0 31 vs 409	144/10/0 10 vs 298	> 0.05	7
				rs12980275	AA/AG/GG G vs A	90/16/0 16 vs 196	208/34/1 36 vs 450	185/18/0 18 vs 388	> 0.05	
				rs8099917	GG/GT/TT G vs T	90/16/0 16 vs 196	215/26/0 26 vs 456	192/12/0 12 vs 396	> 0.05	
Seto WK [28]	2013/China	Asian	Hospital-based	rs12979860	T vs C	–	11 vs 192	14 vs 189	> 0.05	7
				rs8099917	G vs T	–	13 vs 190	15 vs 188	> 0.05	
Senem CK [29]	2014/Turkey	Caucasian	Population-based	rs12979860	CC/CT/TT T vs C	–	46/65/6 77 vs 157	215/201/57 315 vs 631	> 0.05	7
				rs12980275	AA/AG/GG G vs A	–	36/67/15 97 vs 139	191/207/58 323 vs 589	> 0.05	
Akkiz H [30]	2014/Turkey	Caucasian	Hospital-based	rs12979860	CC/CT/TT T vs C	92/91/25 141 vs 275	–	43/50/17 84 vs 136	0.30	7
Liao Y [31]	2014/ China	Asian	Hospital-based	rs12979860	CC/CT + TT T vs C	–	420/66 66 vs 906	391/66 70 vs 844	> 0.05	8
				rs12980275	AA/AG + GG G vs A	–	418/68 68 vs 904	388/69 77 vs 842	> 0.05	
				rs8099917	GG/GT + TT G vs T	–	470/16 16 vs 956	433/24 24 vs 890	> 0.05	
Shi XD [32]	2015/China	Asian	Population-based	rs12979860	CC/CT/TT T vs C	19/0/0 0 vs 38	–	114/23/0 23 vs 251	> 0.05	7
Kimkong [33]	2015/Thailand	Asian	Hospital-based	rs12979860	CC/CT/TT T vs C	–	140/24/0 24 vs 304	116/12/0 12 vs 244	> 0.05	7
				rs8099917	GG/GT/TT G vs T	–	0/25/139 25 vs 303	0/8/120 8 vs 248	> 0.05	
Song YZ [14]	2017/China	Asian	Hospital-based	rs12979860	CC/CT/TT T vs C	400/57/3 63 vs 857	–	439/52/2 56 vs 930	0.79	7
				rs12980275	AA/AG/GG G vs A	398/58/4 66 vs 854	–	437/55/1 57 vs 929	0.50	
				rs8099917	GG/GT/TT G vs T	2/52/406 56 vs 864	–	1/49/443 51 vs 935	> 0.05	
Baghbani JM [34]	2017/Iran	Caucasian	Hospital-based	rs12979860	CC/CT/TT T vs C	–	28/49/17 83 vs 105	31/44/8 60 vs 106	> 0.05	7
				rs8099917	GG/GT/TT G vs T	–	9/32/53 50 vs 138	4/17/62 25 vs 141	> 0.05	

### Association of *IL28B* polymorphisms with HBV persistence

**Fig. 2:**
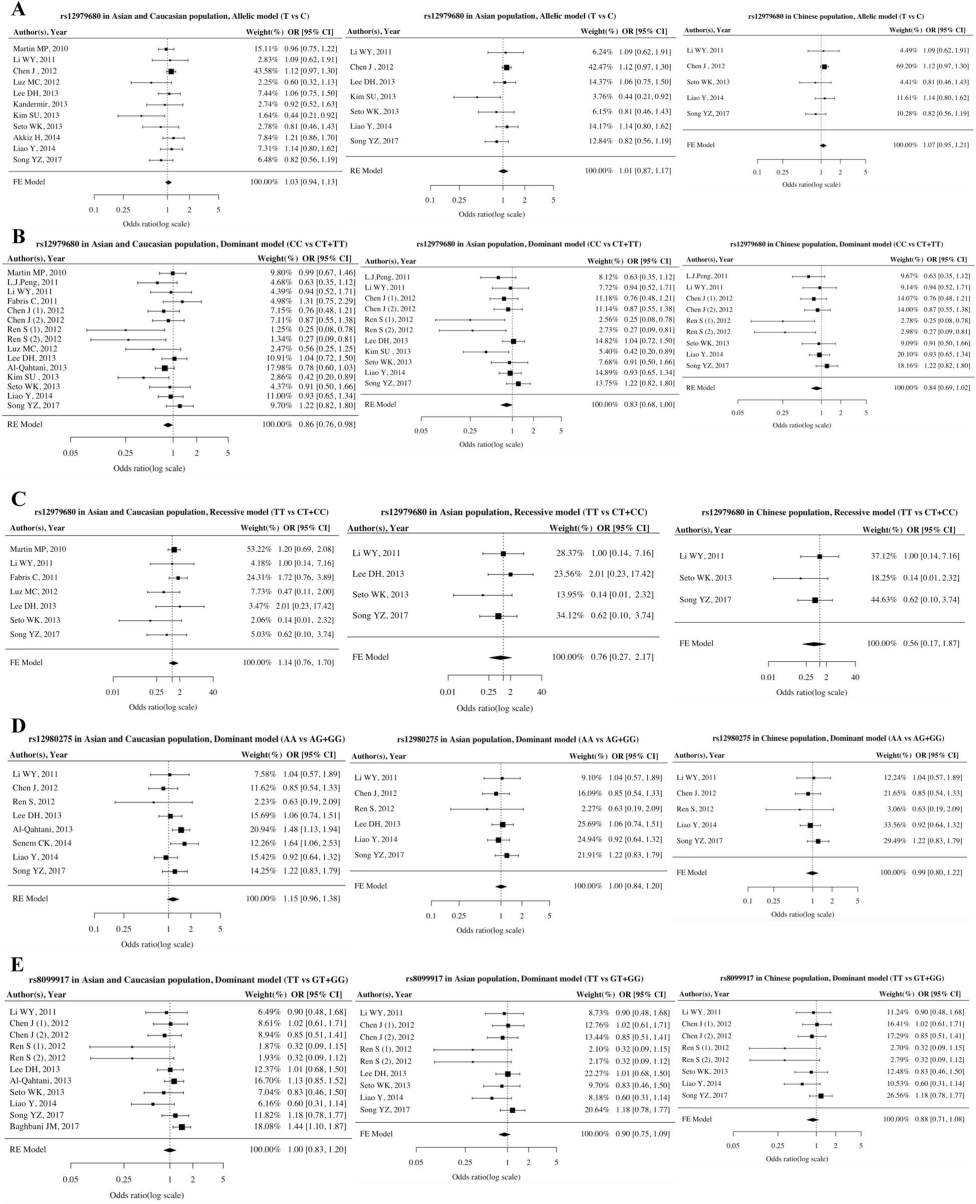
Forest plots of association between *IL28B* polymorphisms and HBV persistence. **a** Overall analysis and subgroup analysis of rs12979860 under the allelic model. **b** Overall analysis and subgroup analysis of rs12979860 under the dominant model. **c** Overall analysis and subgroup analysis of rs12979860 under the recessive model. **d** Overall analysis and subgroup analysis of rs12980275 under the dominant model. **e** Overall analysis and subgroup analysis of rs8099917 under the dominant model

The association between *IL28B* polymorphisms and HBV infection are shown in Fig. 2. There was no association of the SNP rs12979860 with the risk of HBV persistence based on the allelic model (T vs C: OR = 1.03, 95% CI = 0.94–1.13, *P* = 0.53), the dominant model (CC vs CT + TT: OR = 0.86, 95% CI = 0.76–1.00, *P* = 0.05), or the recessive model (TT vs CT + CC: OR = 1.14, 95% CI = 0.76–1.70, *P* = 0.53). As few studies provided sufficient information on rs12980275 and rs8099917, these 2 SNPs were only considered in a dominant model. There was no association of these genotypes with HBV persistent infection (rs12980275 in AA vs AG + GG: OR = 1.15, 95% CI = 0.96–1.38, *P* = 0.17; rs8099917 in TT vs GT + GG: OR = 1.00, 95% CI = 0.83–1.20, *P* = 0.99).

Subgroup analysis was performed for Asian and Chinese populations. There was no evidence that *IL28B* polymorphisms (rs12979860, rs12980275 and rs8099917) were associated with persistent HBV infection in Asian or Chinese populations. For Asians, rs12979860 had no association with HBV infection in the allelic, dominant or recessive model (T vs C: OR = 1.01, 95% CI = 0.87–1.17, *P* = 0.86; CC vs CT + TT: OR = 0.83, 95% CI = 0.68–1.00, *P* = 0.05; TT vs CT + CC: OR = 0.76, 95% CI = 0.27–2.17, *P* = 0.61). SNPs rs12980275 and rs8099917 also had no association with HBV infection in Asians using a fixed-effects model (rs12980275 in AA vs AG + GG: OR = 1.00, 95% CI = 0.84–1.20, *P* = 0.99; rs8099917 in TT vs GT + GG: OR = 0.90, 95% CI = 0.75–1.09, *P* = 0.28). In Chinese, none of these 3 SNPs were associated with HBV persistence (rs12979860 in T vs C: OR = 1.07, 95% CI = 0.95–1.21, *P* = 0.25; CC vs CT + TT: OR = 0.84, 95% CI = 0.69–1.02, *P* = 0.10; TT vs CT + CC: OR = 0.56, 95% CI = 0.17–1.87, *P* = 0.35; rs12980275 in AA vs AG + GG: OR = 0.98, 95% CI = 0.80–1.22, *P* = 0.89; rs8099917 in TT vs GT + GG: OR = 0.87, 95% CI = 0.71–1.08, *P* = 0.28) (Table 2).

**Table 2:** Overall meta-analysis of the association of *IL28B* polymorphisms with persistent HBV infection

SNP	Model	Ethnicity	Studies Number	OR		Heterogeneity			Publication bias		OR_S_
				OR (95% CI)	*P*_OR_	*I*^*2*^ (%)	*P*_H_	M	*P*_Begg_	*P*_Egger_	
rs12979860	Allelic	Total	11	1.03 (0.94, 1.13)	0.528	24.6	0.210	F	0.010	0.015	1.06 (0.97, 1.17)
		Asian	7	1.01 (0.87, 1.17)	0.863	33.1	0.175	R	0.069	0.032	1.07 (0.96, 1.21)
		Chinese	5	1.07 (0.95,1.21)	0.245	0.0	0.476	F	0.233	0.237	Not necessary
	Dominant	Total	15	0.86 (0.76, 1.00)	0.050	37.2	0.073	R	0.016	0.003	0.91 (0.78, 1.06)
		Asian	11	0.83 (0.68, 1.00)	0.051	44.7	0.053	R	0.006	< 0.001	0.95 (0.73, 1.26)
		Chinese	9	0.84 (0.69, 1.02)	0.081	40.8	0.095	R	0.045	0.001	0.98 (0.76, 1.26)
	Recessive	Total	7	1.14 (0.76, 1.70)	0.526	0.0	0.504	F	0.562	0.200	Not necessary
		Asian	4	0.76 (0.27, 2.17)	0.611	0.0	0.513	F	1.000	0.415	Not necessary
		Chinese	3	0.56 (0.17, 1.87)	0.350	0.0	0.526	F	1.000	0.327	Not necessary
rs12980275	Dominant	Total	8	1.15 (0.96, 1.38)	0.135	32.5	0.168	R	0.399	0.177	Not necessary
		Asian	6	1.00 (0.84, 1.20)	0.966	0.0	0.795	F	0.469	0.453	Not necessary
		Chinese	5	0.98 (0.80, 1.22)	0.890	0.0	0.689	F	0.483	0.495	Not necessary
rs8099917	Dominant	Total	11	1.00 (0.83, 1.20)	0.992	41.2	0.074	R	0.002	< 0.001	1.15 (0.96, 1.39)
		Asian	9	0.90 (0.75, 1.09)	0.284	11.0	0.343	F	0.006	0.006	0.99 (0.83, 1.18)
		Chinese	8	0.87 (0.71, 1.08)	0.214	18.5	0.283	F	0.0141	0.006	0.98 (0.81, 1.19)

*SNP* single nucleotide polymorphism, *M* model for meta-analysis, *F* Fixed-effects model, *R* Random-effects model, P_H_*P*-value for heterogeneity test, P_OR_*P*-value for OR test, P_Begg_*P-*value for Begg’s test, *P*_*Egger*_*P*-value for Egger’s test, *OR*_*S*_ OR derived from trim and fill sensitive analysis

### Association of *IL28B* polymorphism rs12979860 with HBV-related HCC

**Fig. 3:**
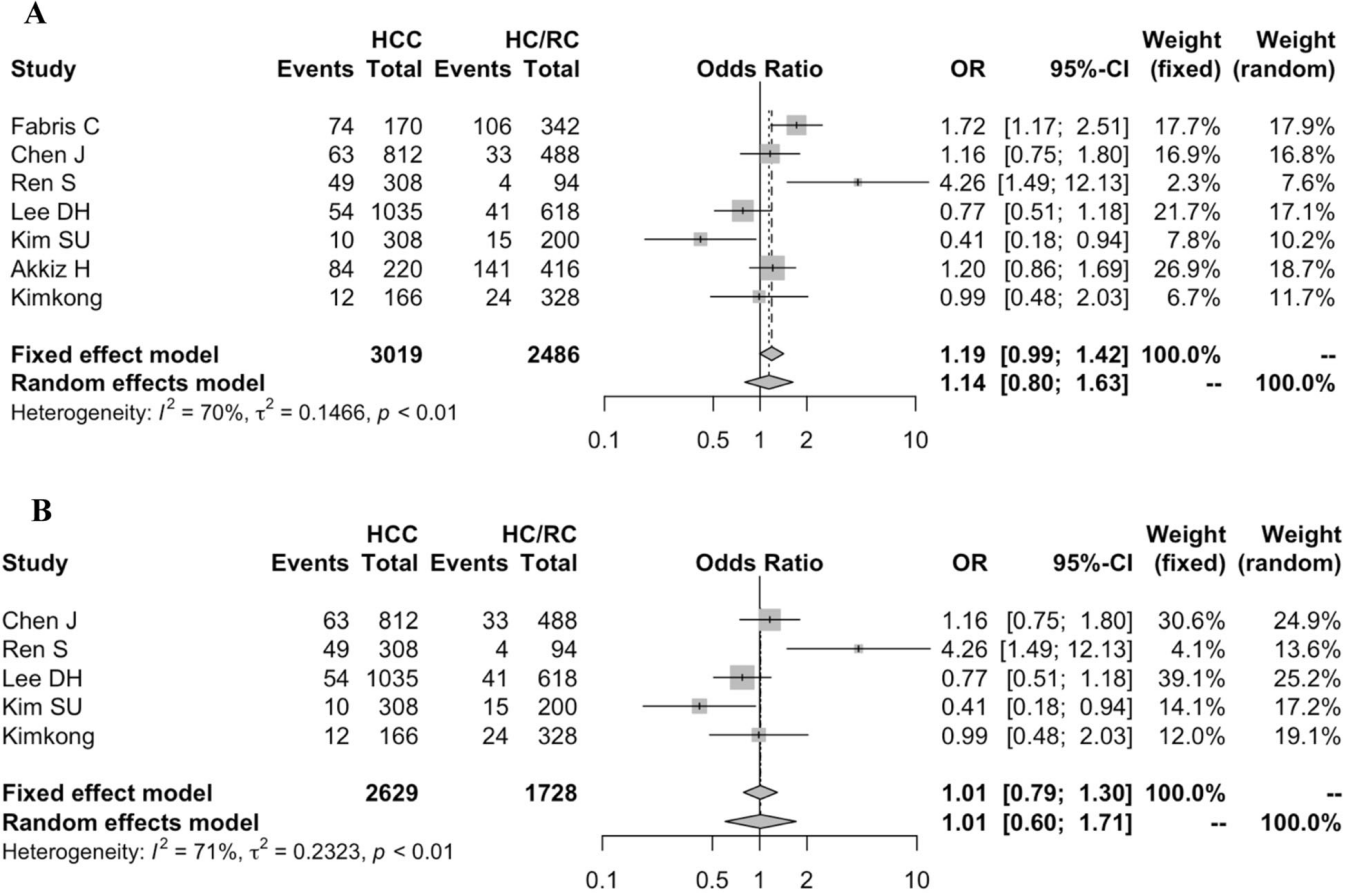
Forest plot for association between *IL28B* rs12979860 polymorphism and the development of HBV-related HCC (T vs C). **a** Overall analysis; **b** Subgroup analysis

HBV-related HCC is the most serious consequence of persistent HBV infection [35]. In order to explore the disease outcome-specific association with genetic factors, we further investigated the association of *IL28B* polymorphisms with the risk of HBV-related HCC. However, few studies provided sufficient information on that, hence We only assess the association of rs12979860 C/T with the development of HBV-related HCC in the allelic model. As shown in Fig. 3, 7 studies comprising 3019 cases and 2486 controls were included in this meta-analysis. A random-effects model was used to estimate the association of the rs12979860 polymorphism with the risk of HCC in HCC patients and healthy controls (HC) or recovered controls (RC). No significant association was observed of the SNP rs12979860 with HBV-related HCC susceptibility (T vs C: OR = 1.14, 95% CI = 0.80–1.63). Subgroup analysis was performed for Asians population, but rs12979860 had no significant association with the development of HBV-related HCC (T vs C: OR = 1.01, 95% CI = 0.60–1.71).

### Sensitivity analysis

#### Association of *IL28B* polymorphisms with HBV persistence

Heterogeneity of the associations between *IL28B* polymorphisms and persistent HBV infection varied from 0 to 44.7%. We found low heterogeneity for the SNP rs12979860 in the allelic and recessive models (T vs C: *I*^*2*^ = 24.6, *P*_H_ = 0.21; TT vs CT + CC: *I*^*2*^ = 0, *P*_H_ = 0.50). However, heterogeneity in the dominant model for *IL28B* polymorphisms (rs12979860, rs12980275 and rs8099917) was significant. Subgroup analyses were carried out to identify sources of heterogeneity. After stratifying by Asian population, there was no heterogeneity for rs12980275 and rs8099917 in the dominant model (rs12980275 in T vs G: *I*^*2*^ = 0, *P*_H_ = 0.80; rs8099917 in A vs G: *I*^*2*^ = 11%, *P*_H_ = 0.34). However, heterogeneity in CC vs CT + TT for rs12979860 was still higher than 25%. We further performed the subgroup analysis stratified by Chinese population, but heterogeneity remained for CC vs CT + TT (*I*^*2*^ = 40.8%, *P*_H_ = 0.095). Sequential omission of individual studies (Additional file 1), after excluding the study of Kim SU [13], the significant pooled OR was no longer significant for CC vs CT + TT (OR = 0.88, 95% CI = 0.75–1.01), but heterogeneity still existed (*I*^*2*^ = 40%). In addition, there was a significant decline in heterogeneity (*I*^*2*^ = 27%), with excluding the study of Ren S [19]. However, it was still higher than 25%.

#### Association of *IL28B* polymorphism rs12979860 with HBV-related HCC

Heterogeneity of these 7 studies was 70%. Sensitivity analysis was performed using the sequential omission approach. However, the heterogeneity remained high, and a random-effects model was used to estimate the pooled ORs. The overall result was not influenced by the sequential omission approach. The 7 pooled ORs and corresponding 95% CIs were presented in Additional file 1.

### Publication Bias

#### Association of *IL28B* polymorphisms with HBV persistence

**Fig. 4:**
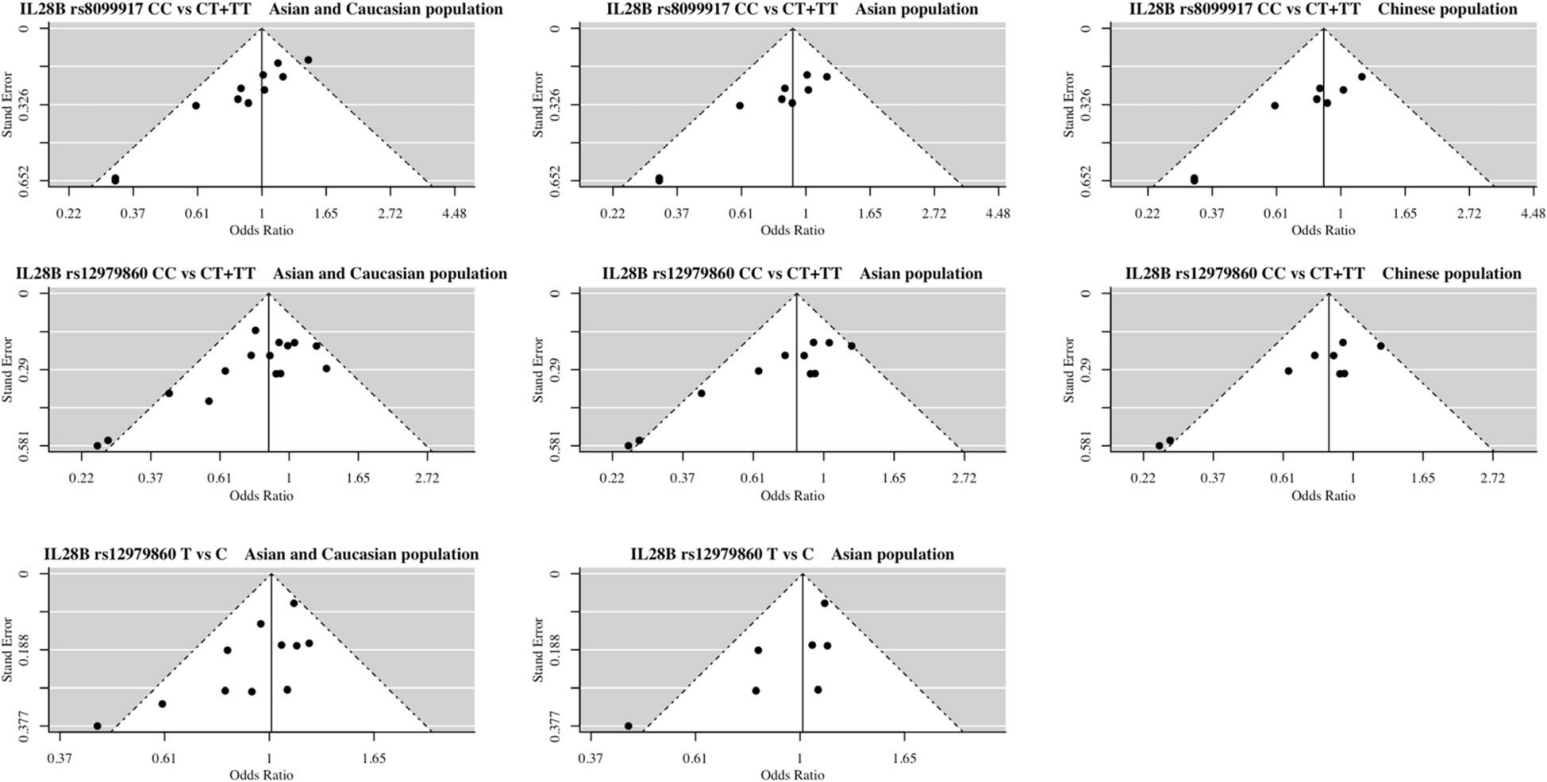
Funnel plot analysis to examine publication bias

In overall and subgroup analysis, publication bias occurred in the allelic and dominant model for rs12979860 and occurred in the dominant model for rs8099917. Begg’s rank correlation, Egger’s weighted regression and trim and fill method were used as the sensitivity analysis to examine the publication bias (Table 2). The presence of publication bias was shown in Fig. 4.

#### Association of *IL28B* polymorphism rs12979860 with HBV-related HCC

There was no obvious asymmetry in the funnel plot for the total population or the Asian population. No publication bias was observed for the association of SNP rs12979860 with HBV-related HCC susceptibility (Total population: *P*_Begg_ = 0.773, *P*_Egger_ = 0.700; Asian population: *P*_Begg_ = 0.817, *P*_Egger_ = 0.400).

## Discussion

In this meta-analysis, we examined the association of *IL28B* polymorphisms with the outcome of HBV infection. We performed a meta-analysis of the associations of *IL28B* polymorphisms (rs12979860, rs12980275 and rs8099917) with HBV persistence. Then we assessed the association of the SNP rs12979860 with the risk of HBV-related HCC. Overall, there was no strong evidence of an association of *IL28B* polymorphisms with HBV infection. As such, these 3 SNPs do not appear to be risk factors for hepatitis B progression. This meta-analysis provides a reliable and comprehensive assessment of the association of *IL28B* polymorphisms with HBV infection. This study is the first systematic review that included all relevant eligible English publications in the recent 8 years. Meanwhile, it had a larger sample size comprising 5587 cases and 4295 than previous studies [36, 37]. Previous studies investigating these associations may have included low-quality publications [38, 39].

Firstly, rs12979860, rs12980275 and rs8099917 had no association with HBV persistence, overall or by subgroup although the heterogeneity was still higher than 25% but was not explained by any individual study. This meta-analysis is consistent with the study of DH Lee [26] showing that *IL28B* polymorphisms are unrelated to HBV infection, assessed from HBsAg seroclearance, rather than the risk of persistent HBV infection and progression of HBV-related HCC. Moreover, according to the strict inclusion criteria we excluded some studies [38, 39] included by Lee. In addition, we included totally 18 recent publications in this meta-analysis, and the quantity was greater than in the 11 publications included in Lee’s study. Similarly, this study is consistent with the study of Tang Sd. et al. [40]. However, there is a difference between that study and this study. In their study, only 9 publications were included and only the rs12979860 C/T polymorphism was investigated to evaluate the relationship. In our study, 18 publications were included to investigate the association of 3 SNPs with HBV persistence. For subgroup analysis, results differed from that in Chen J’s study [24]. They found a significant association of rs12979680 with the risk of persistent HBV infection among Chinese population under an additive model. However, we found no such association in either an allelic, dominant or recessive model. This difference might have occurred because of sample size. There were 406 cases and 244 controls included in Chen J’s study. Smaller studies may have a higher chance of false positives. A published study with small sample size and significant result is more likely to be a false positive because of the low sensitivity [41]. Meanwhile, there was no general principle of gene model selection [42]. However, it is easier to apply logistic regression analysis to dominant and recessive model, which would be more precise to estimate the pooled OR [42].

*IFN*-λ3 is coded by the *IL28B* gene and has an antiviral effect on chronic HCV [43]. Many studies [44–47] have shown an association of *IL28B* polymorphisms with HCV infection. However, the nucleotide analogs may have an additional pharmacological effect by inducing *IFN*-λ3 production, which facilitates interferon-stimulated genes and decreases the production of HBsAg [48]. Moreover, HBV and HCV share a similar natural history, pathogenesis and transmission modality. In order to provide novel insights for HBV treatment, focus has been on the association of *IL28B* polymorphisms with HBV infection [49]. Although several studies [24, 27, 32] have demonstrated *IL28B* polymorphisms predict HBV infection. There is a possibility of producing a false positive result. In the study of Martin MP [20], despite they revealed that the SNP rs12979860 was associated with spontaneous clearance of HBV, this significant association was merely found among one-fifth of patients who were also chronic HCV carriers and 69% patients with HIV coinfection. Therefore, we excluded participants testing positive for anti-HCV or anti-HIV, because the correlation of these diseases with *IL28B* genes would potentially increase the likelihood of false positive in our estimate.

Secondly, rs12979860 had no association with the risk of HBV-related HCC, overall or by subgroup. No publication bias was found by either Begg’s rank correlation method or Egger’s weighted regression method. Nevertheless, this estimate might be underpowered due to the limited number of studies. We obtained consistent findings with a few studies [15, 17, 26]. However, this meta-analysis failed to reach a consensus with the studies of Fabris and Ren S [19, 23]. In the study of Fabris, the researchers found rs12979860 polymorphism contributed to the risk of HCC among chronic HCV patients. However, they did not observe the association of the rs12979860 polymorphism with HBV-related HCC. That might explain the discrepancy. GWAS have identified the *IL28B* gene as a major determinant of the course of HCV infection, and experimental evidence indicates that *IFN*-λ3 coded by *IL28B* gene has an anti-tumor effect [50, 51]. The SNP rs12979860 C/T might only have significant association with the risk of HCV-related HCC. In the study of Ren S, they found a significant association between rs12979860 and HBV-related HCC in the Chinese population. However, we obtained an inconsistent result based on the combined evidence. As HBV involves a complicated interplay between host and virus, no single molecular analysis was expected to fully unravel the disease mechanism. Multiple molecular levels could interact and also show plasticity in different physiological conditions and disease stages [52]. Hence there is a great requirement for additional novel approaches that could combine data from different molecular levels and could help to determine the causal path from genotype to phenotype.

Another thing we would highlight about the HBV-related HCC, is that it is thought to involve the interaction between environmental factors and inherited susceptibility [53]. For instance, recent case-control studies found a positive association between African dietary iron overload and HBV/HCV-related HCC [54–56]. The underlying mechanism could be excessive free irons generating harmful “reactive oxygen species” (ROS) under aerobic conditions in the human body. ROS could damage the balance between oxidative and reductive events, and consequently destroy DNA in hepatocytes and induce HCC [57]. This finding requires further investigation due to the limitation of observational studies. However, dietary iron overload might increase the risk of developing HBV-relate HCC in Africans and might disguise the potential association of the *IL28B* gene with the disease.

This study is a candidate gene association study which is different from GWAS. Candidate gene studies are relatively cheap and quick to perform, and are concentrated on the selection of a putative candidate genetic variants according to its relevance in the mechanism of the disease investigated. However, candidate gene studies are susceptible to publication bias, and they are underpowered due to high rates of false positive findings and low rates of replication [58]. In contrast, GWAS are more reliable and comprehensive approach for identifying loci in the genome that might be relevant to the interest disease. Millions of SNPs in the genome are assessed for genotype-phenotype association. However, the cost of genotyping for a GWAS is at least an order of magnitude higher than the cost to genotype several candidate genes [59]. Hence, candidate gene studies are a cost-effective approach to identify genetic risk factors in the absence of GWAS for a specific disease. Currently, no GWAS has assessed the association of *IL28B* with HBV infection. The unknown history of HBV exposure in the controls or relatively small sample size of the study reduces power to identify genetic variants with modest effect [60].

Conventionally, the association between genotype variants and disease in candidate gene studies was discovered from case-control studies. However, there might be a spurious correlation despite the observed statistical significance. Candidate gene studies are prone to false positive findings for the following reasons. Firstly, the allele may be in linkage disequilibrium with an allele at another locus that directly affects the expression of the phenotype. Secondly, the allele itself is functional and directly affects the expression of the phenotype [61]. Thirdly, differences in the frequency of genetic variants within the population might lead to false positive results. In this study, the minor allele frequencies of these 3 SNPs in Asians were significantly lower than those of the Caucasians, suggesting the presence of population specificity [62]. Lastly, some systemic technical bias would be generated due to different DNA samples from cases and controls, and eventually induced a false positive relationship [63].

There were also several advantages that would further strengthen this study compared to previous similar studies. Above all, the publications we included in this meta-analysis were high-quality based on the NOS score, which increased the internal validity. Moreover, we followed the strict inclusion criteria such as no-coinfection with HCV or HIV, which decreased the bias generated by correlations of these diseases with *IL28B* polymorphisms. Thirdly, this meta-analysis included a total of 5587 cases and 4295, comprising 4913 cases and 3865 controls for rs12979860 C/T, 3765 cases and 2506 controls for rs12980275 A/G, and 3912 cases and 2900 controls for rs8099917 T/G, which were even more than twice the subjects included in previous studies. In addition, we performed a more comprehensive subgroup analysis by ethnicity, because of potential differences between populations. Lastly, this study is the first systematic review that included all relevant eligible English publications in the recent 8 years. Systematic reviews provide a synthesis of evidence for clinical decisions. Hence having an up-to-date and comprehensive review is imperative.

The findings of this systematic review and meta-analysis were limited by several factors. Firstly, the study design included only case-control studies. However, genetic case-control studies are susceptible to selection bias. The key requirement for a case-control study to avoid selection bias is that the cases and controls are selected from the same underlying population, which is difficult to ascertain. Thirdly, the genotype distribution deviated from HWE in 1 study, which might be attributed to population stratification or potential selection bias. Fourthly, we did not have original data for all studies to account for potential confounding however, genetic case control studies are not open to confounding, because few factors are determinants of genetic make-up. Fifthly, the assay methods used to characterize the polymorphisms differed in these studies, i.e. the sensitivity and specificity of identifying *IL28B* polymorphisms were different, which might reduce the reliability. Lastly, although we included 18 studies in this meta-analysis, the presence of heterogeneity and publication bias indicated potential issue. There was still a great need for large, high methodological quality publications from a genome wide association study in both Caucasian and Asian populations to verify our findings.

## Conclusion

Genetic epidemiology is a vital composition of public health research. However, spurious associations might also generate inefficient treatments and waste medical resources. This study is a cost-effective approach to investigating the association of *IL28B* with HBV infection, in the absence of GWAS for this specific association. However, there were still several limitations that might influence the effect of genetic factors on disease outcome. Further well-designed large-scale studies, especially related to the gene-ethnic group and phenotype-environmental interaction are warranted to confirm the real contribution of these polymorphisms to the outcome of HBV infection.

## Supplementary information

**Additional file 1: Supplement 1.** Minor allele frequencies of *IL28B* polymorphisms in persistent HBV infection patients. **Supplement 2.** Heterogeneity analysis of each *IL28B* polymorphism.

## Data Availability

All data generated or analysed during this study are included in this published article and its supplementary information files.

## Abbreviations

IL 28B: Interleukin (IL) 28B
HBV: Hepatitis B virus
SNP: Single nucleotide polymorphism
HIV: Human immunodeficiency virus
HCV: Hepatitis C virus
HHC: Hepatitis hepatocellular carcinoma
HCC: Hepatocellular carcinoma
DALYs: Disability-adjusted life-years
IFN-λ3: Interferon-λ3
GWAS: Genome-wide association studies
PEG-IFN-α: Pegylated interferon-α
OR: Odds ratio
CI: Confidence interval
HBsAg: Hepatitis B surface antigen
anti-HBc: Hepatitis B core antibody
anti-HBs: Hepatitis B surface antibody
HWE: Hardy-Weinberg equilibrium
NOS: Newcastle-Ottawa Scale
HC: Healthy control
RC: Recovered control
