# Supplementary material for: Association between IL28B Polymorphisms and Outcomes of Hepatitis B Virus Infection: A meta-analysis
Source: BMC Med Genet. 2020 May 1;21:88. doi: 10.1186/s12881-020-01026-w (PMC7195703; doi:10.1186/s12881-020-01026-w)
Supplement: Supplementary file 1 — Additional file 1: Supplement 1. Minor allele frequencies of IL28B polymorphisms in persistent HBV infection patients. Supplement 2. Heterogeneity analysis of each IL28B polymorphism. [file 12881_2020_1026_MOESM1_ESM.docx]

**Supplements**

**Supplement** **1** Minor allele frequencies of *IL28B* polymorphisms in persistent HBV infection patients

| SNP | Minor allele^a^/ referent | Ethnicity | Country | Minor allele frequency | China vs other country | |
| --- | --- | --- | --- | --- | --- | --- |
|  |  |  |  |  | *P*^b^ | F_ST_ |
| rs12979860 | T / C | Asian | China | 0.071 | - | - |
|  |  |  | South Korea | 0.060 | 0.220 | 1.504 |
|  |  |  | Thailand | 0.047 | 0.142 | 2.161 |
|  |  | Caucasian | Italy | 0.287 | 0.000 | 85.083 |
|  |  |  | Iran | 0.361 | 0.000 | 159.460 |
|  |  |  | Saudi Arabia | 0.281 | 0.000 | 323.880 |
|  |  |  | Spain | 0.235 | 0.000 | 35.032 |
|  |  |  | Turkey | 0.333 | 0.000 | 374.790 |
|  |  |  | United States | 0.354 | 0.000 | 296.450 |
| rs12980275 | G / A | Asian | China | 0.074 | - | - |
|  |  |  | South Korea | 0.068 | 0.521 | 0.412 |
|  |  | Caucasian | Saudi Arabia | 0.193 | 0.000 | 129.620 |
|  |  |  | Turkey | 0.354 | 0.000 | 406.390 |
| rs8099917 | G / T | Asian | China | 0.054 | - | - |
|  |  |  | South Korea | 0.054 | 0.993 | 0.000 |
|  |  |  | Thailand | 0.076 | 0.118 | 2.443 |
|  |  | Caucasian | Iran | 0.151 | 0.000 | 25.572 |
|  |  |  | Saudi Arabia | 0.151 | 0.000 | 108.720 |

a. Minor allele defined as the minor allele in Chinese Han population.

b. *P*-value calculated by Fisher’s exact test.

**Supplement 2** Heterogeneity analysis of each *IL28B* polymorphism

| Excluding literature | OR_1_ (95% CI) | | | | | | |  | OR_2_ (95% CI) | |
| --- | --- | --- | --- | --- | --- | --- | --- | --- | --- | --- |
|  |  | rs12979860 |  |  | rs12980275 |  | rs8099917 | |  | rs12979860 |
|  | Allelic | Dominant | Recessive |  | Dominant |  | Dominant | |  | Allelic |
| None | 1.03 (0.94, 1.13) | 0.91 (0.78, 1.06) | 1.14 (0.76, 1.70) |  | 1.15 (0.96, 1.38) |  | 1.15 (0.96, 1.39) | |  | 1.14 (0.80, 1.63) |
| Martin MP | 1.01 (0.88, 1.15) | 0.84 (0.73, 0.98) | 1.07 (0.60, 1.93) |  | - |  | - | |  | - |
| L.J.Peng | - | 0.88 (0.77, 1.00) | - |  | - |  | - | |  | - |
| Li WY | 1.00 (0.89, 1.13) | 0.86 (0.74, 0.98) | 1.15 (0.76, 1.73) |  | 1.16 (0.95, 1.41) |  | 1.00 (0.83, 1.21) | |  | - |
| Fabris C | - | 0.85 (0.74, 0.96) | 1.00 (0.63, 1.59) |  | - |  | - | |  | 1.04 (0.71, 1.54) |
| Chen J | 0.96 (0.85, 1.09) | 0.87 (0.76, 1.00) | - |  | 1.20 (1.00, 1.45) |  | 0.98 (0.80, 1.20) | |  | 1.14 (0.74, 1.76) |
| Ren S | - | 0.88 (0.78, 0.99) | - |  | 1.17 (0.97, 1.41) |  | 1.03 (0.87, 1.23) | |  | 1.04 (0.75, 1.43) |
| Luz MC | 1.03 (0.94, 1.15) | 0.87 (0.77, 0.99) | 1.23 (0.81, 1.87) |  |  |  | - | |  | - |
| Lee DH | 0.99 (0.87, 1.13) | 0.84 (0.73, 0.97) | 1.12 (0.74, 1.68) |  | 1.16 (0.94, 1.44) |  | 0.98 (0.79, 1.21) | |  | 1.23 (0.84, 1.82) |
| Al-Qahtani | 1.01 (0.90, 1.13) | 0.87 (0.75, 1.01) | - |  | 1.08 (0.91, 1.28) |  | 0.95 (0.77, 1.18) | |  | - |
| Kim SU | 1.04 (0.95, 1.15) | 0.88 (0.78, 1.00) | - |  | - |  | - | |  | 1.26 (0.91, 1.75) |
| Seto WK | 1.02 (0.92, 1.14) | 0.86 (0.75, 0.98) | 1.19 (0.79, 1.79) |  | - |  | 1.01 (0.84, 1.22) | |  | - |
| Senem CK | - | - | - |  | 1.13 (0.97, 1.32) |  | - | |  | - |
| Akkiz H | 0.97 (0.86, 1.11) | - | - |  | - |  | - | |  | 1.13 (0.71, 1.79) |
| Liao Y | 0.99 (0.87, 1.12) | 0.85 (0.73, 0.98) | - |  | 1.20 (0.99, 1.46) |  | 1.05 (0.89, 1.25) | |  | - |
| Shi XD | - | - | - |  | - |  | - | |  | - |
| Kimkong | - | - | - |  | - |  | - | |  | 1.16 (0.78, 1.73) |
| Song YZ | 1.04 (0.94, 1.15) | 0.83 (0.73, 0.95) | 1.18 (0.78, 1.78) |  | 1.13 (0.91, 1.41) |  | 0.96 (0.78, 1.18) | |  | - |
| Baghbani JM | - | - | - |  | - |  | 0.96 (0.82,1.13) | |  | - |

OR_1_: The association of *IL28B* polymorphisms with the risk of HBV persistence; OR_2_: The association of rs12979860 with the risk of HBV-related HCC.
